# Genomic Characterization and Phylogenetic Classification of Bovine Coronaviruses Through Whole Genome Sequence Analysis

**DOI:** 10.3390/v12020183

**Published:** 2020-02-06

**Authors:** Tohru Suzuki, Yoshihiro Otake, Satoko Uchimoto, Ayako Hasebe, Yusuke Goto

**Affiliations:** 1 Division of Viral Disease and Epidemiology, National Institute of Animal Health, National Agriculture and Food Research Organization, Tsukuba, Ibaraki 3050856, Japan; 2 Central Tochigi Prefectural Livestock Health and Hygiene Center, Utsunomiya, Tochigi 3210905, Japan; lovedropzera@gmail.com; 3 Shiga Prefectural Livestock Health and Hygiene Center, Omihachiman, Shiga 5230813, Japan; uchimoto-satoko@pref.shiga.lg.jp; 4 Central Gifu Prefectural Livestock Health and Hygiene Center, Gifu 5011112, Japan; hasebe-ayako@pref.gifu.lg.jp; 5 Central Iwate Prefectural Livestock Health and Hygiene Center, Takizawa, Iwate 0200605, Japan; y.gotou@pref.iwate.jp

**Keywords:** bovine coronavirus, whole genome, spike protein, phylogenetic analysis, genotype classification

## Abstract

Bovine coronavirus (BCoV) is zoonotically transmissible among species, since BCoV-like viruses have been detected in wild ruminants and humans. BCoV causing enteric and respiratory disease is widespread in cattle farms worldwide; however, limited information is available regarding the molecular characterization of BCoV because of its large genome size, despite its significant economic impact. This study aimed to better understand the genomic characterization and evolutionary dynamics of BCoV via comparative sequence and phylogenetic analyses through whole genome sequence analysis using 67 BCoV isolates collected throughout Japan from 2006 to 2017. On comparing the genomic sequences of the 67 BCoVs, genetic variations were detected in 5 of 10 open reading frames (ORFs) in the BCoV genome. Phylogenetic analysis using whole genomes from the 67 Japanese BCoV isolates in addition to those from 16 reference BCoV strains, revealed the existence of two major genotypes (classical and US wild ruminant genotypes). All Japanese BCoV isolates originated from the US wild ruminant genotype, and they tended to form the same clusters based on the year and farm of collection, not the disease type. Phylogenetic trees on hemagglutinin-esterase protein (HE), spike glycoprotein (S), nucleocapsid protein (N) genes and ORF1 revealed clusters similar to that on whole genome, suggesting that the evolution of BCoVs may be closely associated with variations in these genes. Furthermore, phylogenetic analysis of BCoV S genes including those of European and Asian BCoVs and human enteric coronavirus along with the Japanese BCoVs revealed that BCoVs differentiated into two major types (European and American types). Moreover, the European and American types were divided into eleven and three genotypes, respectively. Our analysis also demonstrated that BCoVs with different genotypes periodically emerged and predominantly circulated within the country. These findings provide useful information to elucidate the detailed molecular characterization of BCoVs, which have spread worldwide. Further genomic analyses of BCoV are essential to deepen the understanding of the evolution of this virus.

## 1. Introduction

Bovine coronavirus (BCoV) causes neonatal calf diarrhea, winter dysentery in adult cattle, and respiratory tract disorders in cattle of all age groups [1,2,3,4,5]. BCoV infections in herds lead to a reduction in milk production, deterioration of health, and deaths of young animals, resulting in severe economic losses [6,7]. In general, there are two different types (enteric and respiratory) of BCoVs based on disease symptoms. Although some previous reports have suggested that several candidate amino acids may contribute to the differences between the two disease types, it still remains unclear [8,9,10]. 

BCoV is a member of the family *Coronaviridae*, genus *Betacoronavirus* [11]. The genome is a single-stranded, positive-sense RNA of approximately 31 kb. It includes 10 open reading frames (ORFs) flanked by 5’and 3’untranslated regions. ORF1a and ORF1b encode polyproteins, which are further cleaved to multiple nonstructural proteins. ORF3, ORF4, ORF8, ORF9, and ORF10 encode hemagglutinin-esterase protein (HE), spike glycoprotein (S), small membrane protein (E), membrane protein (M), and nucleocapsid protein (N), respectively, which are components of the virion. The remaining ORFs encode unknown or less-characterized proteins. 

The S glycoprotein is cleaved into S1 (N terminus) and S2 (C terminus) subunits by an intracellular protease [12,13]. The S1 subunit mediates interactions with specific cellular receptors, and induces neutralizing antibodies and hemagglutination activity [14,15,16,17,18]. However, the S2 subunit is required to mediate fusion of viral and cellular membranes [19,20]. Although the S2 subunit is highly conserved among BCoVs, the S1 subunit is more variable, and mutations in this region are closely associated with altered antigenicity and pathogenicity [21,22,23,24].

Previous studies have reported a high prevalence of BCoV infections in cattle herds in many countries [7,25,26,27,28,29]. Furthermore, BCoV-like coronaviruses are present in various wild ruminants [30,31,32,33]. Moreover, BCoV-like human enteric coronavirus (HCoV-4408/USA/1994) has been previously isolated from a child with acute diarrhea [34]. Public health concerns between ruminants and humans warrant resolution to further the current understanding of the genetic diversity and evolution of BCoV. 

Previous molecular phylogenetic analyses have targeted the S genes of BCoVs detected worldwide [12,26,29,35,36,37,38,39]. Kanno et al. [27,40] reported the prevalence of and genetic variations among BCoVs collected in past decades in Japan. However, few studies have analyzed the genetic diversity and evolution of BCoVs based on entire genomes, not a specific gene. In the present study, we attempted to further deepen the current understanding of the evolutionary dynamics of BCoVs by comparing whole genomes from BCoVs collected in Japan during 2006–2017 and reference BCoVs available in GenBank. Furthermore, we attempted to identify specific genetic markers to distinguish the two disease types by comparing complete sequences of the spike glycoproteins from these BCoVs.

## 2. Materials and Methods

### 2.1. Samples and Ethics Statement

From 2006 to 2017, 92 fecal samples and/or nasal swabs were collected from cattle of all age groups exhibiting diarrhea and/or respiratory disorders at 54 farms in Japan by authorized veterinarians on owner’s consent. Subsequently, 67 BCoVs were isolated from these samples by using human rectal tumor (HRT-18) cell culture in four relevant Livestock Health and Hygiene Service Centers, respectively (Table S1). As samples were collected as a part of a routine diagnostic procedure, permission regarding animal ethics was not required. 

### 2.2. RNA Extraction and Sequence Analysis

Viral RNA was extracted from supernatants using QIAamp Viral RNA Mini Kit (Qiagen, Venlo, Limburg, The Netherlands) in accordance with the manufacturer’s instructions. The cDNA of BCoVs was synthesized using the PrimeScript High Fidelity RT-PCR Kit (Takara, Shiga, Japan) with a random 6-mer primer in accordance with the manufacturer’s instructions. The whole genome comprising six overlapping amplicons (approximately 5 kb) except for 22 nt at the 5’end and 54 nt at the 3’end with reference to the Kakegawa strain, a Japanese classical strain collected in 1972, was generated from cDNA, using sets of primers originally designed with reference to the reference strain (Table S2). Six amplicons were pooled in equal amounts and analyzed using a next-generation sequencer (Ion Torrent PGM; Thermo Fisher Scientific, Carlsbad, CA, USA). The consensus full-genome sequences of the 67 BCoV isolates were determined with reference to the complete genome of the Kakegawa strain. Whole genome sequences determined herein were submitted to the DNA Data Bank of Japan (DDBJ), and are retrievable from GenBank. The GenBank accession numbers (LC494126-LC494192) for each BCoV isolate are summarized in Table S1. 

### 2.3. Phylogenetic Analysis

Sequences were aligned using Clustal W method in the MEGA X software [41]. Genetic distances for the whole genomes and individual ORFs were calculated using the Kimura two-parameter correction at the nucleotide level. Phylogenetic analyses using whole genomes and individual ORFs from 83 strains were performed by the maximum-likelihood method with the general time reversible nucleotide substitution model and 1000 bootstrap replicates, using the MEGA X program [41]. Moreover, phylogenetic analysis using the S genes of 153 BCoV strains, by adding sequences of multiple BCoV strains from Denmark, Sweden, Germany, Italy, France, and Korea, and BCoV-like human coronavirus (HCoV-4408) to those of the 83 BCoV strains, was also conducted using the aforementioned method. Genotype classification of whole genomes and S genes was conducted using cut-off values calculated based on the definition that was used in genotype classification of rotavirus [42,43]. Briefly, the cut-off values of whole genomes and S genes were estimated as the percentage separating the intra-genotype identities (the nucleotide identities among strains belonging to the same genotype), and the inter-genotype identities (the nucleotide identities among strains belonging to different genotypes). However, in cases that inter- and intra-genotype identities partially overlapped, the most appropriate cut-off value was chosen as the percentage at which the ratio of the inter-genotype identity and intra-genotype identity dropped below 1.

## 3. Results

### 3.1. Sequence Analysis

The nearly full-length genome sequences from 67 BCoV isolates were determined by two-step RT-PCR using a set of primers designed in this study with reference to the complete genome of the Kakegawa strain (Table S3). ORF1 sequences from 41 BCoV isolates and the IWT-14 isolate were 21281 and 21269 nucleotides (nt) in length, different from the length of those (21284 nt) of the 25 remaining BCoV isolates and the reference strain. The ORFs of the S genes from 65 BCoV isolates had the same length (4092 nt) as the reference strain; however, the sequences of the two remaining BCoV isolates (TCG-17 and TCG-18) were 4086 nt with a 6-nt deletion in the S1 subunit. The lengths of ORF5 sequences from 42 and 24 BCoV isolates were 81 and 90 nt, respectively, differing markedly from that (132 nt) of the reference strain. In addition, ORF5 sequence from the one remaining isolate (SHG-3) was 120 nt in length, which was 12-nt shorter than that of the reference strain. The ORF6 sequences from ten BCoV isolates (TCG-24 to TCG-33) were 129 nt in length, being shorter than those (138 nt) of the 57 remaining BCoV isolates and the reference strain. The ORFs of the N genes from 58 BCoV isolates had the same length (1347 nt) as that of the reference strain, differing from the length of those (1338 nt) from the eight BCoV isolates (IWT-7, IWT-8, SHG-1, SHG-2, TCG-10, TCG-11, TCG-12, and TCG-14) and that (1344 nt) of the IWT-27 isolate. Nucleotide sequences of the 5 remaining ORFs had the same length among 67 BCoV isolates and were identical to those of the reference strain. 

### 3.2. Whole Genome Phylogenetic Analysis

**Figure 1:**
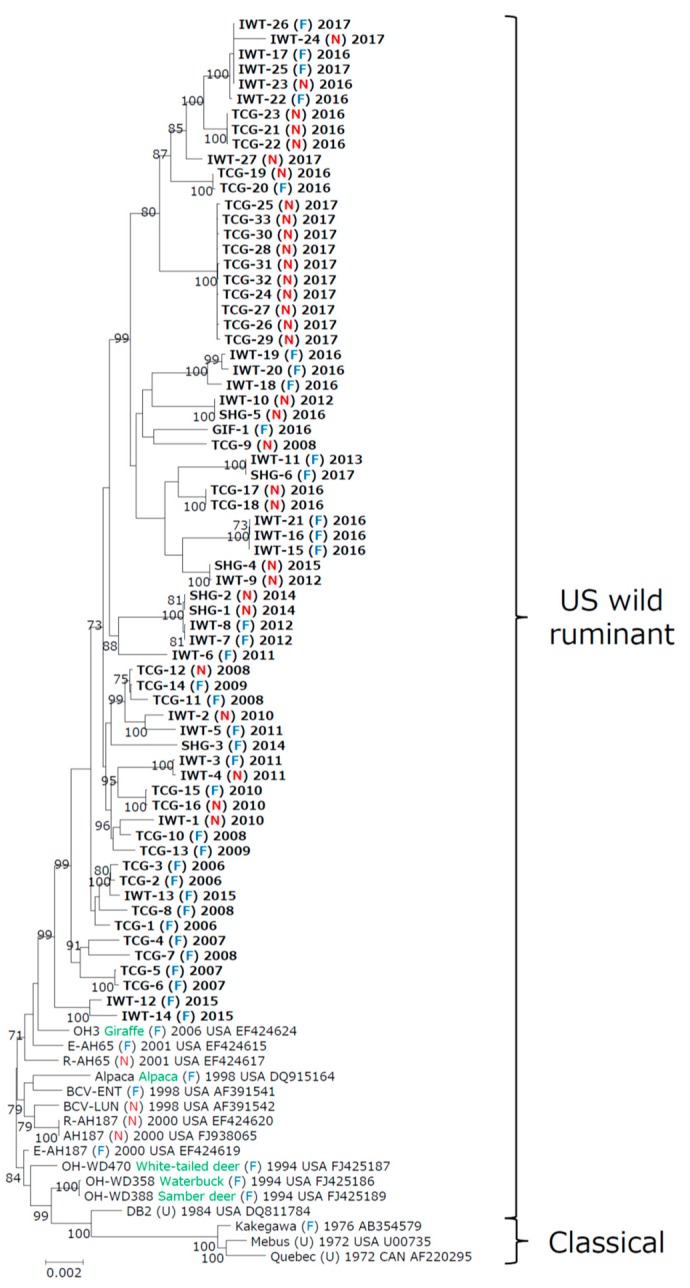
Phylogenetic tree constructed using whole genomes of 67 recent bovine coronavirus (BCoV) isolates from Japan and 16 reference BCoV strains from other countries. The tree was constructed using the maximum-likelihood method with the general time reversible nucleotide substitution model implemented in the MEGA X program. Numbers at each branch represent groups with >70% bootstrap support using 1000 replicates. Strain name, host (green): in case of wild ruminant, source: fecal sample (F), nasal swab (N), and unknown (U), year of collection, country, and the GenBank accession number are indicated. Bold text represents the Japanese BCoV isolates used in this study. The scale bar indicates nucleotide substitutions per site.

Phylogenetic analyses using whole genomes and individual ORFs were performed by adding data from 67 Japanese BCoV isolates to those of 16 BCoV strains available in GenBank. On whole genome phylogenetic analysis, 83 BCoV strains were classified into two major genotypes with a cut-off value of 98.8%, viz., classical and US wild ruminant genotype, of which all Japanese BCoV isolates were widely derived from the US wild ruminant genotype (Figure 1 and Table 1). Surprisingly, the 67 Japanese BCoV isolates formed random clusters, not specific clusters, depending on two disease types. Of them, there were several clusters showing existence of the two disease types between isolates derived from the same farm such as IWT-3 and 4, TCG-11 and 12, TCG-15 and 16, and TCG-19 and 20. In contrast, there were mainly three types upon grouping the 67 BCoV isolates based on farm and year of collection. They included type I (farm of collection is the same, and year of collection is the same): IWT-18, 19, and 20, SHG-1 and 2, TCG-2 and 3, TCG-17 and 18, TCG-21, 22, and 23, and TCG-24 to TCG-33; type II (farm of collection is different, but year of collection is the same): IWT-7 and 8, IWT-15, 16, and 21, IWT-17 and IWT-22 to IWT-26, TCG-5 and 6, and TCG-10 and 13; and type III (farm of collection is different, and year of collection is different): IWT-9 and SHG-4, IWT-10 and SHG-5, and IWT-11 and SHG-6. Taken together, phylogenetic analysis using whole genomes from the 67 BCoV isolates revealed that the isolates tended to form clusters corresponding to year and/or farm of collection, and not the disease type.

Moreover, phylogenetic analyses for HE, S, and N genes encoding structural proteins and ORF1 encoding nonstructural proteins yielded a similar classification to that using whole genome with each of the estimated cut-off values (Table 1). 

### 3.3. Phylogenetic Analysis for S Genes

**Figure 2:**
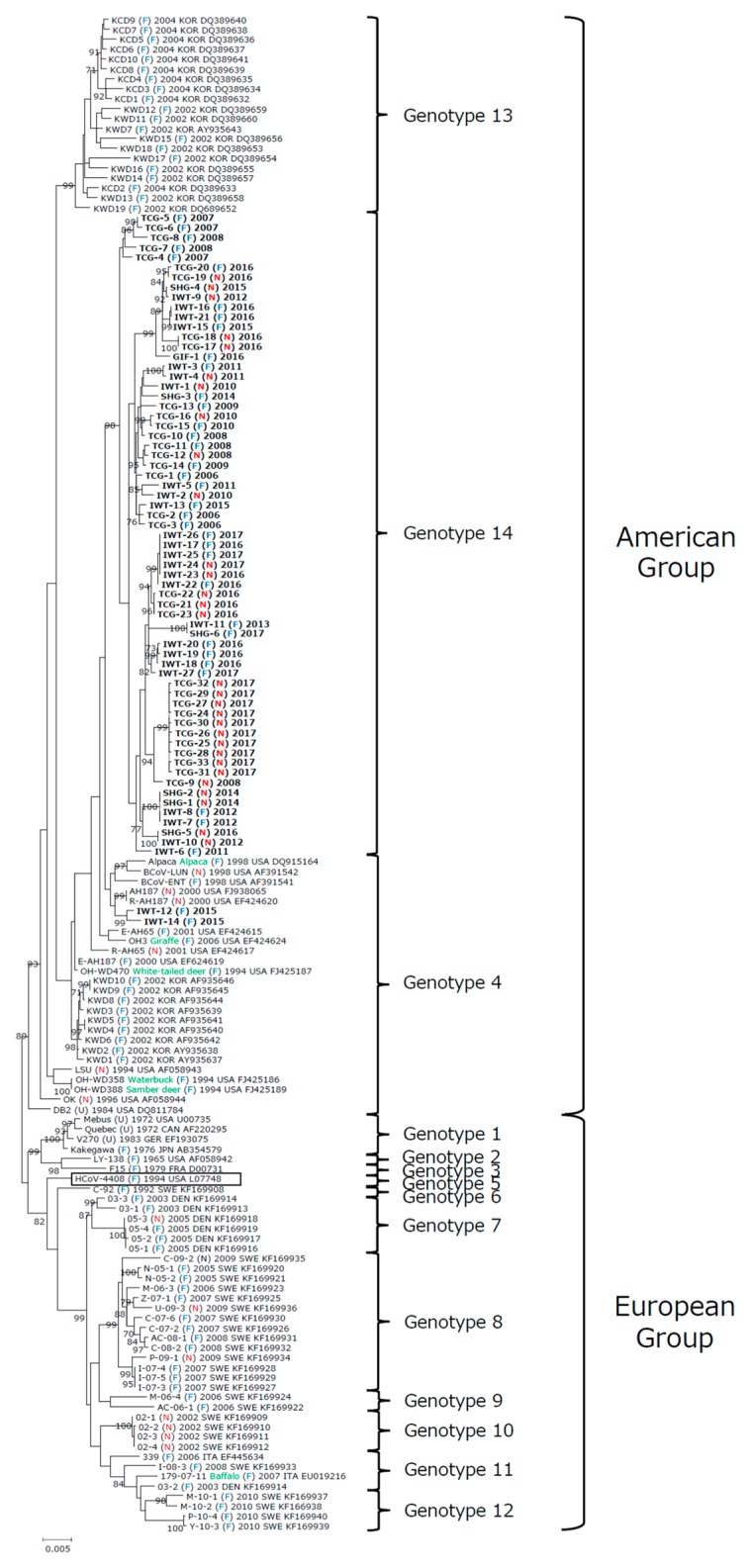
Phylogenetic tree constructed using complete sequences of spike glycoproteins from 67 recent BCoV isolates collected from Japan, previously reported BCoV strains collected in the United States, Canada, Sweden, Denmark, Italy, Germany, France, and Korea, and human enteric coronavirus (HCoV-4408). The tree was constructed using the maximum-likelihood method with the general time reversible nucleotide substitution model implemented in the MEGA X program. Numbers at each branch represent groups with >70% bootstrap support using 1000 replicates. Strain name, host (green): in case of wild ruminant, source: fecal sample (F), nasal swab (N), and unknown (U), year of collection, country, and the GenBank accession number are indicated. Bold text represents the Japanese BCoV isolates used in this study. Box represents human enteric coronavirus. The scale bar indicates nucleotide substitutions per site.

To better understand the evolutionary dynamics of BCoVs, we also performed phylogenetic analysis using complete sequences of the spike glycoproteins from American, European, and Asian BCoV strains in addition to the recent Japanese BCoV isolates. Consequently, 153 BCoV strains were classified into two major types: European type including Swedish and Danish BCoV strains derived from the classical genotype and American type including Korean and Japanese BCoV strains derived from the US wild ruminant genotype (Figure 2). In addition, the European and American types were divided into eleven and three genotypes with a cut-off value of 99.0% calculated on the basis of the definition that was used in genotype classification of rotavirus (Table 2): Genotype 1, four classical BCoV strains by adding V270 to three classical BCoV strains; Genotype 2, one classical BCoV strain from USA (LY138); Genotype 3, one classical BCoV strain from France (F15); Genogroup 4, fifteen American BCoV strains including BCoV-like strains from wild ruminants, nine Korean BCoV strains causing winter dysentery from 2002, and two recent Japanese BCoV isolates (IWT-12 and IWT-14); Genogroup 5, BCoV-like human enteric coronavirus (HCoV-4408); Genogroup 6, one Swedish BCoV strain from 1992 (C-92); Genogroup 7, six Danish BCoV strains from 2003 and 2005; Genogroup 8, fourteen Swedish BCoV strains from 2005 to 2009; Genogroup 9, two Swedish BCoV strains from 2006; Genogroup 10, four Swedish BCoV strains from 2002; Genotype 11, three BCoV strains from Sweden, Italy, and Denmark and one BCoV-like strain from Buffalo; Genotype 12, four Swedish BCoV strains from 2010; Genotype 13, twenty Korean BCoV strains causing calf diarrhea and winter dysentery from 2002 and 2004; and Genotype 14, sixty-five recent Japanese BCoV isolates. The recent Japanese BCoV isolates belonged to two genotypes (genotypes 4 and 14). Moreover, the BCoV strains collected in the same year and/or the same country tended to formed one cluster.

## 4. Discussion

Herein, we determined the whole genome sequences of 67 BCoV isolates collected from 46 farms throughout Japan, using a next generation sequencer based on methods we previously developed to analyze other coronaviruses, porcine epidemic diarrhea virus, and porcine deltacoronavirus [44,45]. Whole genomes sequence analysis of the 67 Japanese BCoV isolates revealed differences in sequence length in the five of ten ORFs encoded by the BCoV genome. Notably, two BCoV isolates (TCG-17 and TCG-18) causing respiratory disorders had 6-nt deletions in the S1 subunit (position 1619–1624) of the S gene. This region partially overlaps with the deletion region (position 1622–1636) of the S gene of BCoV-like strain, OH3 isolated from a giraffe calf [32]. Thus, these data suggest that this region might be involved in the function of spike protein. Our results show that the eight BCoV isolates (IWT-7, IWT-8, SHG-1, SHG-2, TCG-10, TCG-11, TCG-12, and TCG-14) and one BCoV isolate (IWT-27) had 9-nt (position 621–629) and 3-nt (position 1200–1202) deletions in the N genes, respectively. The coronavirus N protein is a multifunctional protein that forms a complex with genomic RNA, interacts with the viral M protein, and enhances the efficacy of viral transcription and assembly; hence, these variations in the N gene might affect the life cycle of the virus [46,47]. In contrast, the effects of differences in the sequences of ORF5 and ORF6 on the mechanism of action of BCoV are unclear, owing to a lack of understanding of their functions.

Comparisons of whole genomes and individual ORFs among the 67 Japanese BCoV isolates and between these isolates and 16 reference BCoV strains revealed the genetic diversity of BCoVs circulating in Japan and worldwide, respectively. Although the highest variation was observed in the ORF5 and ORF6, whose functions are unclear, relatively high variations were also detected in the HE, S, and N genes. Furthermore, genetic variations in the S and HE sequences were similar to those described previously [29]. 

Phylogenetic analyses using whole genomes and individual ORFs of 16 reference BCoV strains and the 67 Japanese BCoV isolates were performed using the maximum-likelihood method with the MEGA X program [41]. Analyses of whole genomes, HE, S, N genes, and ORF1 encoding important factors regulating viral replication and transcription revealed that the 83 BCoV strains were similarly differentiated into two major genotypes. The recent Japanese BCoV isolates were widely derived from the US wild ruminant genotype of two genotypes. These findings along with comparative sequence analysis revealed that the evolution of BCoVs is probably closely associated with the diversity of these genes. The 67 Japanese BCoV isolates formed a cluster based on the farm and year of collection, but not disease type, showing that the new virus is periodically emerged and widespread in Japan, strongly supporting the results of a molecular study of BCoV in Japan [27,40]. 

In this study, we aimed to understand the current evolutionary dynamics of BCoVs through whole genome analysis of multiple Japanese BCoV isolates collected in recent years. However, our analysis showed that all recent Japanese BCoV isolates and other BCoV and BCoV-like strains collected in the U.S., except for classical BCoV strains, were classified into one same cluster. This fact suggests that it is still difficult to understand the genetic diversity and evolution of BCoVs, which have been spread worldwide, because of limitation in available genomic information and collected countries. Therefore, further whole genome analysis of BCoVs from other countries including European and Asian countries will be required to monitor and prevent the spread of BCoVs, which are transmitted between wild ruminants and humans.

Furthermore, we performed phylogenetic analysis for the S gene, which has been analyzed previously, to understand the evolutionary dynamics of BCoVs in detail [12,26,29,35,36,37,38,39]. The analysis using 153 American, European, and Asian BCoVs including BCoV-like human enteric coronavirus showed the existence of two major types. Moreover, these two types were differentiated into 14 genotypes with calculated cut-off value based on the definition that was used in genotype classification of rotaviruses [42,43]. The European type is derived from the classical genotype, comprising 11 genotypes including Swedish, Danish, German, Italian, and French BCoVs, and HCoV-4408. In contrast, the American type is derived from the US wild ruminant genotype, comprising 3 genotypes including American, Korean, and Japanese BCoVs. Our analysis was performed using data from American and Asian BCoVs together with European BCoVs, therefore we proposed the presence of 14 genotypes when classifying the S gene, compared to previous studies [29,35]. Our analysis revealed that different BCoVs exist, depending on country and year of collection. This finding would be strongly supported by a continuous further analysis of BCoVs.

We also aimed to identify specific genetic markers to distinguish the two disease types by comparing complete sequences of the spike glycoproteins from the recent 67 Japanese BCoV isolates. Unexpectedly, however, all Japanese BCoVs collected in recent years, except for IWT-12 and 14, were classified into one genotype consisting of multiple clusters, which could not be differentiated depending on enteric and respiratory type. We also found that several pairs of isolates were derived from the two disease types in the same farm. Furthermore, these clusters predominantly originated from isolates collected in the same year rather than disease type. Therefore, these findings suggest the possibility that enteric and respiratory disorders caused by BCoVs may occur depending on circumstances such as the health condition of the host, the environment wherein cattle are reared, and collection timing of sources, with no influence of the specific genetic region. In future study, we will need to analyze the correlation of disease occurrence with other genes including the S gene, while accumulating whole genomes of BCoVs from the two different types, in order to detect a genetic marker closely related to these disease types.

In conclusion, this study partially evaluated the current evolutionary dynamic properties of BCoV via comparative and phylogenetic analyses using multiple whole genome sequences by a next generation sequencing-based approach. Our results provide basic information to study the molecular epidemiology of BCoV in future. Moreover, our attempt potentially encourages the establishment of a powerful tool to address public health concerns regarding BCoVs, which have been transmitted between wild ruminants and humans in recent years. 

## Figures and Tables

**Table 1:** Sequence identities (%) of whole genomes and individual open reading frames among classical genotypes from 3 BCoV strains (Quebec, Mebus, and Kakegawa), and US wild ruminant genotypes from the 80 remaining BCoV strains including 67 Japanese BCoV isolates, and between classical and US wild ruminant genotypes at nucleotide level.

	Whole Genome	ORF1	ORF2	ORF3 (HE)	ORF4 (S)	ORF5	ORF6	ORF7	ORF8 (E)	ORF9 (M)	ORF10 (N)
Among classical genotypes	99.7–99.8	99.6–99.8	99.5–99.6	99.7–99.9	99.6–99.8	100	98.4–100	99.4–99.7	99.2–99.6	99.6–100	99.6–100
Among US wild ruminant genotypes (Among 67 Japanese BCoV isolates)	98.8–100 (99.0–100)	99.1–100 (99.1–100)	97.7–100 (97.7–100)	98.1–100 (98.5–100)	98.2–100 (98.4–100)	87.5–100 (90.5–100)	91.2–100 (91.2–100)	97.8–100 (97.8–100)	97.6–100 (98.0–100)	98.4–100 (98.7–100)	98.3–100 (98.3–100)
Between classical and US wild ruminant genotypes	98.2–99.2	98.4–99.3	96.9–98.5	96.9–98.6	97.4–98.9	89.1–98.7	85.6–95.8	97.5–98.4	98.0–100	97.9–99.4	97.5–99.1
Cut-off value	98.8	99.1	98.0	98.3	98.3	ND	ND	97.8	ND	98.6	98.4

ND: Not determined.

**Table 2:** Sequence identities (%) among 14 genotypes that classified using complete sequences of spike glycoprotein (S) genes from 153 American, European, and Asian BCoV strains including BCoV-like human enteric coronavirus at nucleotide level.

Geno-Types ^a^	1 (*n* = 4)	2 (*n* =1)	3 (*n* =1)	4 (*n* = 26)	5 (*n* = 1)	6 (*n* = 1)	7 (*n* = 6)	8 (*n* = 14)	9 (*n* = 2)	10 (*n* = 4)	11 (*n* = 4)	12 (*n* = 4)	13 (*n* = 20)	14 (*n* = 65)
1	99.6–99.8	98.6–98.8	98.2–98.5	97.9–98.9	98.4–98.7	98.1–98.3	97.6–98.1	97.3–98.0	97.5–97.8	97.6–97.9	97.6–98.0	97.4–97.7	97.6–98.5	97.4–98.1
2			98.8	97.9–98.6	98.5	98.1	97.9–98.2	97.6–98.0	97.8–97.9	97.8–97.9	97.8–98.1	97.6–97.7	97.7–98.4	97.5–98.0
3				97.7–98.3	98.2	97.9	97.4–97.8	97.1–97.6	97.3–97.5	97.5	97.4–97.6	97.2–97.3	97.3–98.0	97.1–97.7
4				98.7–100	98.2–98.8	98.1–98.7	97.7–98.7	97.3–98.4	97.6–98.2	97.7–98.4	97.7–98.4	97.4–98.1	97.9–99.2	98.2–99.4
5						99.0	98.3–98.7	97.9–98.4	98.1	98.3–98.4	98.1–98.4	98.0–98.1	97.7–98.5	97.7–98.2
6							98.4–98.8	97.9–98.4	98.1	98.5	98.1–98.6	98.0–98.1	97.6–98.3	97.6–98.1
7							99.0–100	98.2–98.9	98.4–98.9	98.6–99.0	98.3–99.1	98.1–98.6	97.4–98.3	97.2–98.1
8								98.9–100	98.2–98.8	98.2–98.8	97.9–99.0	98.0–98.5	97.0–98.1	96.9–97.9
9									98.7	98.4–98.5	98.3–98.9	98.4–98.6	97.3–97.9	97.0–97.7
10										100	98.6–99.0	98.2–98.4	97.5–98.2	97.2–97.8
11											98.9–99.4	98.4–99.2	97.3–98.2	97.1–97.9
12												98.8–99.0	97.1–97.9	97.0–97.6
13													98.5–99.8	97.5–98.6
14														98.7–100

^a^ Each genotype includes the strains shown in Figure 2.

## Supplementary Materials

The following are available online at https://www.mdpi.com/1999-4915/12/2/183/s1, Table S1: Summary of individual information regarding 67 BCoV isolates collected in Japan from 2006 to 2017, Table S2: List of primers originally designed with reference to the Kakegawa strain (GenBank accession number: AB354579), Table S3: Summary of individual open reading frame (in length) and whole genome (in length) from 67 BCoV isolates collected in Japan from 2006 to 2017.
