# Supplementary material for: Genomic Characterization and Phylogenetic Classification of Bovine Coronaviruses Through Whole Genome Sequence Analysis
Source: Viruses. 2020 Feb 6;12(2):183. doi: 10.3390/v12020183 (PMC7077292; doi:10.3390/v12020183)
Supplement: Supplementary file 1 [file viruses-12-00183-s001.pdf]

Table S1. Summary of individual information regarding 67 BCoV isolates collected in Japan from 2006 to 2017

Strain	Farm	Cattle type	Age	Collection date	Source	GenBank accession number
TCG-1	A	Dairy	Breeding	2006.11	Fecal sample	LC494164
TCG-2	B	Beef	Unknown	2006.12	Fecal sample	LC494163
TCG-3	B	Beef	Unknown	2006.12	Fecal sample	LC494162
TCG-4	C	Beef	Fattening	2007.11	Fecal sample	LC494160
TCG-5	D	Beef	Adult	2007.12	Fecal sample	LC494161
TCG-6	E	Beef	Adult	2007.12	Fecal sample	LC494172
TCG-7	F	Dairy	Unknown	2008.1	Fecal sample	LC494173
TCG-8	G	Beef	Fattening	2008.1	Fecal sample	LC494174
TCG-9	H	Dairy	Calf	2008.3	Nasal swab	LC494177
TCG-10	I	Dairy	Unknown	2008.12	Fecal sample	LC494165
TCG-11	J	Dairy	Unknown	2008.12	Fecal sample	LC494166
TCG-12	J	Dairy	Unknown	2008.12	Nasal swab	LC494167
TCG-13	K	Dairy	Adult	2009.1	Fecal sample	LC494168
TCG-14	L	Dairy	Adult	2009.3	Fecal sample	LC494169
TCG-15	M	Dairy	Adult	2010.1	Fecal sample	LC494170
TCG-16	M	Dairy	Adult	2010.1	Nasal swab	LC494171
TCG-17	N	Dairy	Calf	2016	Nasal swab	LC494176
TCG-18	N	Dairy	Calf	2016	Nasal swab	LC494175
TCG-19	O	Dairy	Calf	2016.12	Nasal swab	LC494178
TCG-20	O	Dairy	Calf	2016.12	Fecal sample	LC494179
TCG-21	O	Dairy	Calf	2016.12	Nasal swab	LC494180
TCG-22	O	Dairy	Calf	2016.12	Nasal swab	LC494181
TCG-23	O	Dairy	Calf	2016.12	Nasal swab	LC494182
TCG-24	O	Dairy	Calf	2017.12	Nasal swab	LC494183
TCG-25	O	Dairy	Calf	2017.12	Nasal swab	LC494184
TCG-26	O	Dairy	Calf	2017.12	Nasal swab	LC494185
TCG-27	O	Dairy	Calf	2017.12	Nasal swab	LC494186

TCG-28	O	Dairy	Calf	2017.12	Nasal swab	LC494187
TCG-29	O	Dairy	Calf	2017.12	Nasal swab	LC494188
TCG-30	O	Dairy	Calf	2017.12	Nasal swab	LC494189
TCG-31	O	Dairy	Calf	2017.12	Nasal swab	LC494190
TCG-32	O	Dairy	Calf	2017.12	Nasal swab	LC494191
TCG-33	O	Dairy	Calf	2017.12	Nasal swab	LC494192
IWT-1	P	Beef	Breeding	2010.10	Nasal swab	LC494129
IWT-2	Q	Beef	Breeding	2010.12	Nasal swab	LC494130
IWT-3	R	Beef	Fattening	2011.6	Fecal sample	LC494131
IWT-4	R	Beef	Fattening	2011.6	Nasal swab	LC494132
IWT-5	S	Dairy	Adult	2011.11	Fecal sample	LC494133
IWT-6	T	Beef	Adult	2011.12	Fecal sample	LC494134
IWT-7	U	Dairy	Adult	2012.1	Fecal sample	LC494135
IWT-8	V	Dairy	Adult	2012.1	Fecal sample	LC494136
IWT-9	W	Beef	Breeding	2012.4	Nasal swab	LC494127
IWT-10	X	Beef	Fattening	2012.5	Nasal swab	LC494128
IWT-11	Y	Dairy	Adult	2013.12	Fecal sample	LC494137
IWT-12	Z	Dairy	Breeding	2015.5	Fecal sample	LC494138
IWT-13	AA	F1 hybrid	Calf	2015.6	Fecal sample	LC494139
IWT-14	AB	Beef	Adult	2015.11	Fecal sample	LC494140
IWT-15	AC	Dairy	Calf	2016.1	Fecal sample	LC494141
IWT-16	AD	Beef	Calf	2016.7	Fecal sample	LC494146
IWT-17	AE	Dairy	Calf	2016.11	Fecal sample	LC494147
IWT-18	AF	Dairy	Unknown	2016.12	Fecal sample	LC494142
IWT-19	AF	Dairy	Unknown	2016.12	Fecal sample	LC494143
IWT-20	AF	Dairy	Unknown	2016.12	Fecal sample	LC494144
IWT-21	AF	Dairy	Unknown	2016.12	Fecal sample	LC494145
IWT-22	AG	Dairy	Adult	2016.12	Fecal sample	LC494148
IWT-23	AH	Beef	Breeding	2016.12	Nasal swab	LC494149
IWT-24	AI	Beef	Breeding	2017.1	Nasal swab	LC494150

IWT-25	AJ	Dairy	Calf	2017.1	Fecal sample	LC494151
IWT-26	AK	Dairy	Adult	2017.2	Fecal sample	LC494152
IWT-27	AL	Dairy	Breeding	2017.2	Nasal swab	LC494153
GIF-1	AM	Beef	Adult	2016.1	Fecal sample	LC494126
SHG-1	AO	Beef	Breeding	2014.9	Nasal swab	LC494154
SHG-2	AO	Beef	Breeding	2014.9	Nasal swab	LC494155
SHG-3	AP	Dairy	Breeding	2014.12	Fecal sample	LC494156
SHG-4	AQ	Beef	Breeding	2015.10	Nasal swab	LC494157
SHG-5	AR	Dairy	Calf	2016.6	Nasal swab	LC494158
SHG-6	AS	Dairy	Adult	2017.10	Fecal sample	LC494159

Table S2. List of primers originally designed with reference to the Kakegawa strain (GenBank accession number: AB354579)

Primers	Sequence (5′ to 3′)	Nucleotide position in Kakegawa strain
Amplicon 1F	TGCATCCCGCTTCACTGATCTCTT	23-46
Amplicon 1R	CACCTACAGGCACAAACCTGTTAGTG	4938-4963
Amplicon 2F	GCACTTGATGATGATGCACGAACC	4672-4695
Amplicon 2R	GGTGGTTGGTAAAGCACATCACTACC	9892-9917
Amplicon 3F	GCCACGCTTTATTTCCCTTGGGAGAT	9502-9527
Amplicon 3R	GAACACCACGTGTAGCTGCTATAC	15037-15060
Amplicon 4F	GAGGAGCAGGATGAAATTTACGCC	14868-14891
Amplicon 4R	ACATCCTGACCCTCTTTACGCACA	19994-20017
Amplicon 5F	GACGTGTGTTGGAAGCACGTCATTTG	19704-19729
Amplicon 5R	GTAAAAACACCTGCAGGTTGAGGC	25023-25046
Amplicon 6F	GTGCGAGTACCCACATACGATTTG	24132-24155
Amplicon 6R	CTGGGTGGTAACTTAACATGCTGG	30961-30984

Table S3. Summary of individual open reading frame (in length) and whole genome (in length) from 67 BCoV isolates collected in Japan from 2006 to 2017.

Strain	ORF1	ORF2	ORF3	ORF4	ORF5	ORF6	ORF7	ORF8	ORF9	ORF10	Full-length
TCG-1	21281	837	1275	4092	90	138	330	255	693	1347	30951
TCG-2	21281	837	1275	4092	90	138	330	255	693	1347	30923
TCG-3	21281	837	1275	4092	90	138	330	255	693	1347	30936
TCG-4	21284	837	1275	4092	90	138	330	255	693	1347	30954
TCG-5	21284	837	1275	4092	90	138	330	255	693	1347	30940
TCG-6	21284	837	1275	4092	90	138	330	255	693	1347	30952
TCG-7	21284	837	1275	4092	90	138	330	255	693	1347	30951
TCG-8	21281	837	1275	4092	90	138	330	255	693	1347	30951
TCG-9	21281	837	1275	4092	90	138	330	255	693	1347	30936
TCG-10	21281	837	1275	4092	81	138	330	255	693	1338	30926
TCG-11	21281	837	1275	4092	81	138	330	255	693	1338	30934
TCG-12	21284	837	1275	4092	81	138	330	255	693	1338	30942
TCG-13	21281	837	1275	4092	81	138	330	255	693	1347	30950
TCG-14	21284	837	1275	4092	81	138	330	255	693	1338	30945
TCG-15	21284	837	1275	4092	81	138	330	255	693	1347	30938
TCG-16	21284	837	1275	4092	81	138	330	255	693	1347	30936
TCG-17	21281	837	1275	4086	81	138	330	255	693	1347	30945
TCG-18	21281	837	1275	4086	81	138	330	255	693	1347	30941
TCG-19/	21281	837	1275	4092	81	138	330	255	693	1347	30949
TCG-20	21281	837	1275	4092	81	138	330	255	693	1347	30936
TCG-21	21281	837	1275	4092	81	138	330	255	693	1347	30939

TCG-22	21281	837	1275	4092	81	138	330	255	693	1347	30923
TCG-23	21281	837	1275	4092	81	138	330	255	693	1347	30940
TCG-24	21281	837	1275	4092	90	129	330	255	693	1347	30936
TCG-25	21281	837	1275	4092	90	129	330	255	693	1347	30940
TCG-26	21281	837	1275	4092	90	129	330	255	693	1347	30942
TCG-27	21281	837	1275	4092	90	129	330	255	693	1347	30942
TCG-28	21281	837	1275	4092	90	129	330	255	693	1347	30940
TCG-29	21281	837	1275	4092	90	129	330	255	693	1347	30942
TCG-30	21281	837	1275	4092	90	129	330	255	693	1347	30938
TCG-31	21281	837	1275	4092	90	129	330	255	693	1347	30899
TCG-32	21281	837	1275	4092	90	129	330	255	693	1347	30942
TCG-33	21281	837	1275	4092	90	129	330	255	693	1347	30942
IWT-1	21281	837	1275	4092	81	138	330	255	693	1347	30934
IWT-2	21284	837	1275	4092	81	138	330	255	693	1347	30954
IWT-3	21284	837	1275	4092	90	138	330	255	693	1347	30955
IWT-4	21284	837	1275	4092	90	138	330	255	693	1347	30954
IWT-5	21284	837	1275	4092	81	138	330	255	693	1347	30954
IWT-6	21281	837	1275	4092	81	138	330	255	693	1347	30951
IWT-7	21281	837	1275	4092	81	138	330	255	693	1338	30945
IWT-8	21281	837	1275	4092	81	138	330	255	693	1338	30941
IWT-9	21284	837	1275	4092	81	138	330	255	693	1347	30951
IWT-10	21284	837	1275	4092	81	138	330	255	693	1347	30954
IWT-11	21281	837	1275	4092	81	138	330	255	693	1347	30951
IWT-12	21284	837	1275	4092	90	138	330	255	693	1347	30954

IWT-13	21281	837	1275	4092	90	138	330	255	693	1347	30942
IWT-14	21269	837	1275	4092	90	138	330	255	693	1347	30939
IWT-15	21284	837	1275	4092	81	138	330	255	693	1347	30950
IWT-16	21284	837	1275	4092	81	138	330	255	693	1347	30954
IWT-17	21281	837	1275	4092	81	138	330	255	693	1347	30944
IWT-18	21284	837	1275	4092	81	138	330	255	693	1347	30954
IWT-19	21284	837	1275	4092	81	138	330	255	693	1347	30954
IWT-20	21284	837	1275	4092	81	138	330	255	693	1347	30954
IWT-21	21284	837	1275	4092	81	138	330	255	693	1347	30954
IWT-22	21281	837	1275	4092	81	138	330	255	693	1347	30946
IWT-23	21281	837	1275	4092	81	138	330	255	693	1347	30944
IWT-24	21284	837	1275	4092	81	138	330	255	693	1347	30954
IWT-25	21281	837	1275	4092	81	138	330	255	693	1347	30951
IWT-26	21281	837	1275	4092	81	138	330	255	693	1347	30950
IWT-27	21281	837	1275	4092	81	138	330	255	693	1344	30944
GIF-1	21281	837	1275	4092	81	138	330	255	693	1347	30951
SHG-1	21281	837	1275	4092	81	138	330	255	693	1338	30936
SHG-2	21281	837	1275	4092	81	138	330	255	693	1338	30938
SHG-3	21284	837	1275	4092	120	138	330	255	693	1347	30942
SHG-4	21284	837	1275	4092	81	138	330	255	693	1347	30953
SHG-5	21284	837	1275	4092	81	138	330	255	693	1347	30950
SHG-6	21281	837	1275	4092	81	138	330	255	693	1347	30947
